# Aspergillus Nodule in a Patient With Autoimmune Pulmonary Alveolar Proteinosis

**DOI:** 10.7759/cureus.29095

**Published:** 2022-09-12

**Authors:** Yuhei Nagaoka, Komiya Kosaku, Hiroki Yoshikawa, Miyuki Abe, Michiyo Miyawaki, Tsutomu Daa, Kazufumi Hiramatsu, Kenji Sugio, Jun-ichi Kadota

**Affiliations:** 1 Respiratory Medicine and Infectious Diseases, Oita University, Yufu, JPN; 2 Respiratory Medicine and Infectious Diseases, Oita University Faculty of Medicine, Yufu, JPN; 3 Thoracic and Breast Surgery, Oita University, Yufu, JPN; 4 Thoracic and Breast Surgery, Oita University, Yuhu city, JPN; 5 Diagnostic Pathology, Oita University, Yufu, JPN

**Keywords:** surgical resection, malignancy, solid nodule, aspergilloma, autoimmune pulmonary alveolar proteinosis

## Abstract

Although autoimmune pulmonary alveolar proteinosis (APAP) is more likely to be associated with infectious diseases, clinical case-based evidence is too limited to confirm this. We describe a case of a man in his late forties diagnosed with APAP nine years prior to the current presentation. A nodule in the right upper lobe gradually increased from 8 to 12 mm over a period of 6 months and was suspicious of malignancy. The pathological analyses revealed *Aspergillus* nodule without any malignant features. This study aims to report a case of *Aspergillus* nodule with APAP and discuss the differential diagnosis of solitary lung nodule developed in APAP.

## Introduction

Pulmonary alveolar proteinosis is an autoimmune disease characterized by macrophage dysfunction [1]. Autoimmune pulmonary alveolar proteinosis (APAP), which is caused by anti-granulocyte-macrophage colony-stimulating factor (GM-CSF) antibodies, accounts for >90% of pulmonary alveolar proteinosis [2]. While APAP is known to be at high risk of infectious diseases, its frequency and etiological microorganism have not been fully investigated. According to a retrospective study by Inoue et al., infectious diseases were complicated in 12 (5.7%) of 212 patients with APAP [3]. Among them, *Aspergillus* infection (1.9%) was the major pathogen, followed by nontuberculous mycobacteria (1.4%) and mycobacterial tuberculosis (0.9%). However, the detailed diagnostic evidence for these infectious diseases has not yet been described.

In our patient, a solitary lung nodule in the upper lobe was newly detected without preceding cavitary lesion in seven years after the diagnosis of APAP, and its size had gradually increased. Pathological analysis after surgical resection revealed *Aspergillus* nodule without any malignant features. This study aims to report a case of *Aspergillus* nodule in a patient with APAP and discuss the differential diagnosis of a solitary nodule in APAP.

## Case presentation

**Figure 1:**
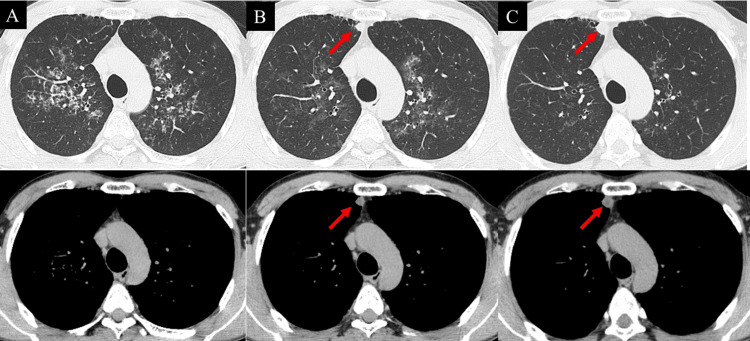
Chest computed tomography features before the appearance of a nodule (A), when a nodule was newly found in the right upper lobe (B), and six months after the detection of the nodule (C). The arrow on each figure indicates the nodule.

A man in his late forties, an ex-smoker (Brinkman index: 420), visited our hospital due to diffuse lung infiltration detected via chest radiography in an annual medical checkup. He was diagnosed with APAP nine years back based on the results of the bronchoalveolar lavage fluid analysis and the positive result for serum GM-CSF antibody. He had occasionally been treated with segmental lung lavage, and his respiratory status was maintained. A solitary nodule newly appeared in the right upper lobe without preceding cavitary or emphysematous lesions on chest high-resolution computed tomography (HRCT) seven years after the diagnosis of APAP (Figures 1A, 1B). Since the size gradually increased from 8 to 12 mm within six months (Figure 1C), he was admitted to the hospital for further evaluation of the lung nodule.

Physical examination upon admission revealed a body temperature of 35.9 °C, oxygen saturation of 98% without supplemental oxygenation, blood pressure of 109/66 mmHg, and heart rate of 53 beats/min. Tumor markers, including cytokeratin 19 fragment and progastrin-releasing peptide, were normal. Cryptococcal antigen nor β-D glucan were normal. A slight uptake of fluorodeoxyglucose was observed in the nodule on positron emission tomography.

**Figure 2:**
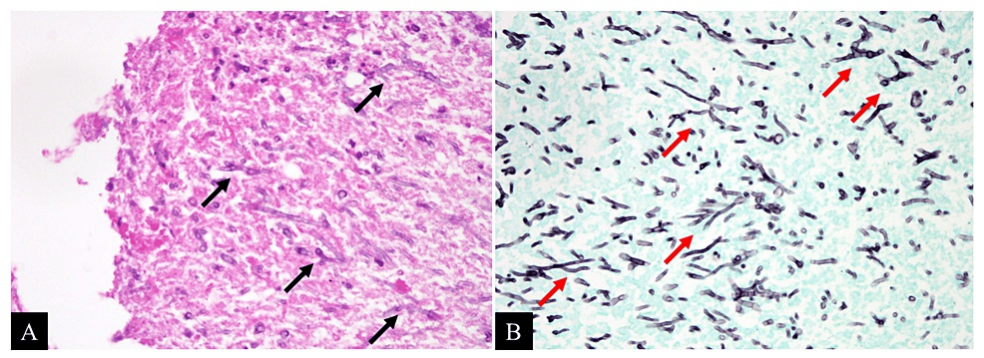
Surgical specimens stained using hematoxylin and eosin (A) and Grocott (B) showing numerous Y-shaped, branching fungal hyphae (arrows).

The nodule was not amenable to biopsy via bronchoscopy based on the anatomy on the HRCT, therefore thoracoscopic partial right lung resection was performed to diagnose the right upper nodule. Since the pathological evaluation revealed numerous Y-shaped branching hyphae in the necrotic material (Figure 2), we diagnosed the patient with *Aspergillus* nodule even though no preceding cavitary or emphysematous lesions were present. He had no adverse effects from the surgery and was discharged 10 days after the operation.

## Discussion

This study reported a case of *Aspergillus* nodule diagnosed using surgical resection in a patient with APAP. It is known that APAP is more likely to be associated with infectious microorganisms, such as fungi, mycobacterium, and nocardiosis, which may present as lung nodules [3-6]. Thus, differential diagnosis considering these diseases along with malignancy is required when a solitary nodule is developed.

*Aspergillus* infection was reported to occur in four (1.9%) of 212 cases in Japanese APAP [3]. However, it is uncertain how many cases of *Aspergillus* nodule accounted for *Aspergillus* infection. Pulmonary nodules are a less common manifestation of *Aspergillus* infection, and *Aspergillus* nodule is likely to develop in patients with emphysematous lesions [7]. However, this case had no preexisting cystic or cavitary lesions. Thus, we could not have suspected of *Aspergillus* nodule and indicated the appropriate surgical resection. The development of *Aspergillus* nodule without preceding cavitary lesions is rarely observed, as only one case has been published by Kurahara [8]. The author reported the case of a patient with aspergilloma (i.e., fungus ball) which developed into nodule-like airspace consolidation in a non-cavitary field. The report described that the patient had no significant underlying disease but provided no information regarding lesion management. Despite the absence of emphysematous lesions, the current patient had a history of smoking, which might be associated with the development of the *Aspergillus *nodule along with APAP. *Aspergillus* nodule is typically diagnosed by precipitating antibodies against *Aspergillus fumigatus* with the corresponding radiological features, but we did not measure the serological maker since *Aspergillus* nodule was not suspected.

A solitary lung nodule is generally considered a malignant, infectious, or noninfectious inflammatory disease. Infectious differential diagnosis of pulmonary nodule includes *Aspergillus* nodule, Coccidioidal nodule, *Histoplasma* nodule, nontuberculous mycobacterial nodule, and *Nocardia* spp. [7]. When the size increases within several months, malignancy needs to be considered. In fact, volume doubling time assessment is advocated for intermediate-sized nodules (diameter of 5-10 mm) [9]. Inoue et al. demonstrated that lung cancer was observed in only one of 212 patients with APAP, which seems to less commonly develop than fungal infections (four of 212) [3]. In this regard, a solitary nodule found in a patient with APAP is more likely to be an infection rather than a malignancy.

## Conclusions

The current case was successfully treated with surgical resection, but some patients with APAP may have deteriorated respiratory function and might not withstand the surgical procedures. Such patients would be benefited if the nodule is appropriately diagnosed, especially with infections, including aspergillosis, *Cryptococcus* infection, or *Actinomycetes* infection, which can be cured with antimicrobial drugs. When a solitary lung nodule appears in a patient with APAP, infectious diseases need to be considered as a potential diagnosis as well as a malignancy.
